# Assessing the impact of antimicrobial stewardship in low-income healthcare settings: a study of antibiotic use and antimicrobial susceptibility patterns in Indian hospitals

**DOI:** 10.1017/ash.2026.10430

**Published:** 2026-06-18

**Authors:** Ulhas Vasave, Amit Paroha

**Affiliations:** Americares India Foundation, India

## Abstract

**Objective:**

The rising incidence of antimicrobial resistance (AMR) underscores the urgent need for effective antimicrobial stewardship (AMS). This study assessed the impact of a tailored AMS initiative in Indian hospitals.

**Methods:**

An AMS surveillance program was implemented across 11 Indian hospitals (January 2022–June 2023). The intervention (July 2022–June 2023) included hospital-specific antibiograms, antibiotic policy design and implementation, monitoring antibiotic consumption, and tracking multidrug-resistant organisms (MDROs). Hospital staff were trained, and compliance audits with feedback were conducted. Implementation followed the Model for Improvement methodology over 18 months.

**Results:**

AMS surveillance revealed significant improvement in AMS knowledge and practices from the baseline to the postintervention phase. Antibiotic compliance increased from a baseline of 10% to 71% across hospitals, reaching 73%–91% in the subsequent period. Surgical prophylaxis compliance improved substantially, particularly in hospitals with initially low adherence. Optimizations in antibiotic selection, dosing, duration, intravenous-to-oral switching, and de-escalation were also observed, resulting in significant compliance gains across all domains. Use of antibiotics classified by the World Health Organization as safer first-line options with a narrow spectrum increased, while use of second- and third-line antibiotics, including those with broader spectra and higher resistance risks, decreased, reflecting a shift toward safer prescribing practices. However, MDRO incidence rates remained variable across sites, showing room for improvement.

**Conclusion:**

Locally tailored AMS interventions within a structured quality improvement framework can establish sustainable practices to mitigate AMR. These findings demonstrate effective strategies for optimizing antibiotic use and provide valuable insights for broader implementation of AMS.

## Introduction

Antimicrobial resistance (AMR) threatens the effectiveness of infection prevention and treatment globally.^1,2^ By 2,050, AMR could cause 4.7 million deaths in Asia alone.^3^ Its drivers include patient misconceptions, indiscriminate prescribing, over-the-counter availability, financial incentives, and physician compliance with patient demands.^1^

Multidrug-resistant organisms (MDROs) are prevalent in 30%–60% of Indian isolates^4^ and include methicillin-resistant *Staphylococcus aureus* (MRSA), vancomycin-resistant enterococci (VRE), carbapenem-resistant Enterobacteriaceae (CRE), and extended-spectrum beta-lactamase (ESBL)-producing gram-negative bacteria, which limit treatment options and increase the risk of untreatable infections.^5^ Resistance, driven by bacterial evolution, can be mitigated through surveillance, antimicrobial stewardship (AMS), and infection prevention and control (IPC) measures.^6^

AMS optimizes antibiotic selection, dosing, and duration, reducing unnecessary or inappropriate prescriptions, which account for 20%–50% of hospital use and contribute to higher mortality and prolonged stays.^7,8^ The WHO Access, Watch, Reserve (AWaRe) framework categorizes antibiotics to manage use and minimize resistance; the 2021 update included 258 antibiotics and emphasized cautious use of reserve drugs, such as colistin.^9,10^

India’s National Action Plan on AMR (2017), aligned with the WHO Global Action Plan, prioritizes strengthening AMS in healthcare facilities.^11^ AMS strategies aim to optimize therapy, reduce resistance, and lower costs.^12^ The 5Ds (Drug, Dose, Delivery Route, Duration, and De-escalation) guide clinicians in optimal antimicrobial therapy.^13^

Despite this, many hospitals have yet to fully implement AMS^14^ Strengthening healthcare professional (HCP) awareness, systematic surveillance, and institutional capacity for AMS programs (AMSPs) is critical for reducing AMR and preserving the effectiveness of existing antimicrobials.^15,16^ This initiative aimed to design and implement an AMSP across multiple Indian hospitals and evaluate outcomes such as policy compliance, antibiotic consumption, and antimicrobial sensitivity, with the goal of proposing a scalable AMS model for wider adoption.

## Methods

### Study design

**Figure 1.**
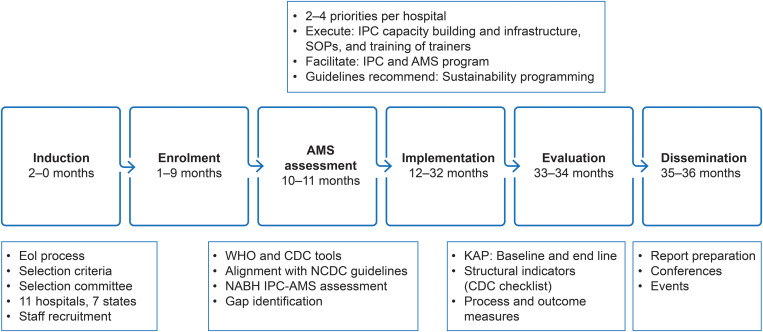
Figure 1 long description. Phases of implementation. AMS, antimicrobial stewardship; CDC, Centers for Disease Control and Prevention; Eol, expression of interest; IPC, infection prevention and control; KAP, knowledge, attitude, and practices; NABH, National Accreditation Board for Hospitals and Healthcare Providers; NCDC: National Center for Disease Control; SOP, standard operating procedure; WHO, World Health Organization.

This study was conducted as part of an initiative to establish and evaluate AMS across selected healthcare facilities in India. A structured workflow was developed to assess baseline AMS status and strengthen institutional capacity for sustainable AMS activities. The study period spanned January 2022 to June 2023 and comprised a 6-month preintervention phase (January–June 2022) and a 12-month intervention phase (July 2022–June 2023). The workflow and interventions were based on the United States Centers for Disease Control and Prevention (US CDC) core elements of hospital AMSPs.^17^ The phases of AMSP implementation are illustrated in Figure 1. This study was designed as a quality improvement initiative and did not involve identifiable personal data, patient recruitment, or deviations from standard care; therefore, ethical approval and informed consent were not required.

### Workflow for AMSP implementation

1. Selection of hospitals: The AMSP was implemented across 11 hospitals in Nagpur, Nashik, Mumbai, Agra, Bagalkot, Jabalpur, Surat, Navi Mumbai, Jaipur, Chennai, and Bhopal. Participating hospitals demonstrated leadership commitment by dedicating human, financial, and technological resources. Hospital selection followed a predefined screening grid that required a minimum bed capacity of 150, availability of multiple specialties, an in-house microbiology laboratory, and an in-house pharmacy with software-enabled antibiotic usage monitoring, operated by qualified microbiologists.
2. Formation of AMS teams: Each hospital established a dedicated AMS team with well-defined roles, including physicians, clinical pharmacists, nurses, microbiologists, information technology support, and hospital leadership, all committed to optimizing antimicrobial use.
3. Baseline assessment: A baseline assessment evaluated the existing antimicrobial consumption data, and the CDC AMS assessment tool was applied to identify gaps.^18^
4. Development of policies and guidelines: The AMS teams developed and disseminated hospital-specific antimicrobial prescribing policies based on local antibiograms to standardize and optimize treatment. These policies addressed key stewardship domains, including adherence to best prescribing practices, optimization of surgical prophylaxis, appropriate antibiotic selection and dosing, IV-to-oral transition, and antibiotic de-escalation. All guidelines were incorporated into hospital AMS manuals to enhance compliance.
5. Education and training (Educate): A structured program trained all HCPs in AMS principles and practices. Comprehensive training programs were implemented to strengthen HCP knowledge and skills in AMS, with an emphasis on current AMS practices and project key performance indicators (KPIs). Quarterly virtual AMS certification courses, conducted in collaboration with the Consortium of Accredited Healthcare Organizations, were incorporated into the intervention framework. Training covered antibiotic policy development, interpretation of microbiology reports, diagnostic stewardship, surgical prophylaxis, pharmacokinetics and pharmacodynamics, molecular principles of antibiotic selection, international AMSP guidelines, the National Action Plan, and implementation challenges. In addition, six self-paced online AMS courses were developed in English and regional languages (Hindi, Marathi, Tamil, Telugu, Malayalam, and Kannada), addressing AMS strategy and surveillance, AMR and susceptibility interpretation, antibiograms and antibiotic policy, MDROs, principles of antibiotic therapy, and antibiotic policy KPIs. These courses were integrated into hospital induction and staff training programs. Training effectiveness was evaluated using pre- and posttraining questionnaires.
6. Implementation of KPIs (Action): To evaluate the program’s impact, predefined KPIs were established across the following outcome measures:
  - AMS assessment: Pre- and postintervention hospital-level scores were evaluated using the CDC assessment tool to track the implementation and maturity of AMS practices. The assessment tool covered the seven CDC core elements of hospital AMSPs, including leadership commitment, accountability, pharmacy expertise, action, tracking, reporting, and education.
  - Knowledge improvement: Knowledge scores were assessed using a structured pre- and posttraining questionnaire administered to participating HCPs. The questionnaire domains were aligned with the AMS training curriculum and included key topics such as antibiotic policy, microbiology interpretation, diagnostic stewardship, surgical prophylaxis, antibiotic selection, AMR/susceptibility interpretation, antibiograms, MDROs, and principles of antibiotic therapy. The questionnaire included multiple-choice items, and scores were expressed as the percentage of correct responses Supplementary Information 1. The assessment was conducted among physicians, pharmacists, nurses, microbiology personnel, and other HCPs involved in AMS activities across the participating hospitals. Across all hospitals, a total of 552 sessions were conducted, and 6,476 HCPs participated.
  - Compliance with AMS practices: To monitor compliance and adherence to policies, as well as support monthly stewardship surveillance, a sample size of 100 patient prescriptions per month per hospital was reviewed using standardized AMS prescription audit tools Supplementary Information 2. These tools were used by the AMS teams, particularly clinical pharmacists and designated stewardship personnel, to review antibiotic prescriptions against hospital-specific guidance and document key stewardship parameters, including indication, antibiotic choice, dose, duration, surgical prophylaxis appropriateness, IV-to-oral switch eligibility, de-escalation based on microbiology results, and use of restricted antibiotics. Audit findings were compiled monthly to assess compliance with AMS practices and support feedback to prescribers. Prescriptions were selected by random sampling from inpatient records involving systemic antibiotic use. Compliance was assessed against hospital-specific antibiotic policies, surgical prophylaxis protocols, and microbiology-guided stewardship recommendations. The following compliance indicators were assessed:
    - Antibiotic compliance rates: Proportion of audited prescriptions that were compliant with the hospital-specific antibiotic policies for indication, agent, dose, route, and duration
    - Surgical prophylaxis compliance: Proportion of audited surgical prophylaxis prescriptions compliant with guidelines for indication, timing, agent choice, and duration
    - Antibiotic selection compliance: Proportion of prescriptions in which the selected antibiotic was appropriate for the documented indication according to hospital guidelines/antibiogram-informed policy
    - Dose optimization: Proportion of prescriptions with dose and frequency consistent with institutional recommendations and clinical context
    - Duration optimization: Proportion of prescriptions with treatment duration consistent with hospital policy or recommended stewardship standards
    - IV-to-oral route conversion rates: Proportion of eligible patients on IV antibiotics who were appropriately transitioned to oral antibiotics
    - De-escalation practices: Proportion of cases in which empiric broad-spectrum antibiotics were narrowed appropriately based on culture results
  - Antibiotic consumption: Antimicrobial consumption was measured monthly using the defined daily dose per 100 bed-days. Usage of WHO-defined “Access,” “Watch,” and “Reserve” antibiotics was monitored.
  - MDRO incidence rates: The incidence of MDROs (MRSA, VRE, ESBL, CRE, and multidrug-resistant [MDR] nonfermenters) was estimated monthly as the number per 1,000 inpatient admissions.
7. Intervention strategies: Priority AMS interventions, including CDC-recommended prospective audits with feedback and antibiotic preauthorization, were implemented to optimize antibiotic use.
  - Surveillance system: A surveillance system was set up to systematically monitor the prescription and administration of antimicrobials and also to analyze patterns of AMR, including MDROs. The hospital’s information system was used for monitoring trends and antimicrobial use patterns.
  - Formulary restrictions and preauthorization: Reserved-category antibiotics (WHO AWaRe) were restricted, and preauthorization was required for broad-spectrum or high-risk use.
  - Prospective audit and feedback: The AMS team audited antimicrobial prescriptions prospectively and provided direct feedback to prescribers, ensuring guideline-based, patient-specific optimization.
  - Antimicrobial timeouts: Scheduled reassessments, known as “antimicrobial timeouts,” were conducted a few days into the course of treatment to evaluate the continued need for or appropriateness of the prescribed antimicrobial therapy. After this period, the antimicrobial drugs were either continued, escalated, or de-escalated based on the patient’s clinical condition. This approach was integrated into the routine prospective audit and feedback system, where the dedicated clinical pharmacist monitored the time lines and consulted the respective clinicians to determine the appropriate timeouts for each patient.
  - Pharmacy-based interventions: Pharmacists played a pivotal role in the AMSP by reviewing antimicrobial orders, ensuring appropriate dosing and duration, and facilitating transitions from IV to oral therapy where applicable. Clinical pharmacists conducted AMS surveillance using audit tools to assess antibiotic policy compliance, antimicrobial consumption, and antibiograms.^19–21^
  - Use of diagnostic tools: The AMSP integrated diagnostic tools to provide accurate identification and susceptibility testing of pathogens. This integration was crucial in allowing timely de-escalation from empiric broad-spectrum antibiotics to more targeted therapies.
  - Celebrate and engage: The AMS team incorporated an element of celebration into their endeavors by orchestrating events such as World Antimicrobial Awareness Week and MRSA Day. These events were designed to stimulate active participation and engagement among hospital staff, reinforcing their commitment to AMSP.
8. Monitoring and sustaining (Tracking and reporting): The AMSP conducted monthly surveillance per NABH standards, with regular team meetings across all tiers. KPI performance was reported to facilitate feedback, consensus, and updates on interventions. Sustainability was promoted by adapting interventions to KPI outcomes and resistance patterns, and project KPIs were communicated through regular newsletters.

### Data collection and analysis

Data were collected from 11 hospitals using prescription audits, direct observation, and patient record reviews to assess adherence to AMS protocols. Compliance rates for each indicator were recorded as baseline and end-of-study period percentages across hospitals. MDRO incidence data were obtained from microbiology laboratory reports and infection surveillance records. Pre- and postintervention indicators were compared to assess the effectiveness of AMSP. The Wilcoxon signed-rank test assessed statistically significant differences from baseline across KPIs. Analyses were conducted using Excel (Microsoft Corporation) and R (version 4.5.0).

## Results

### AMS assessment and knowledge improvement

**Figure 2.**
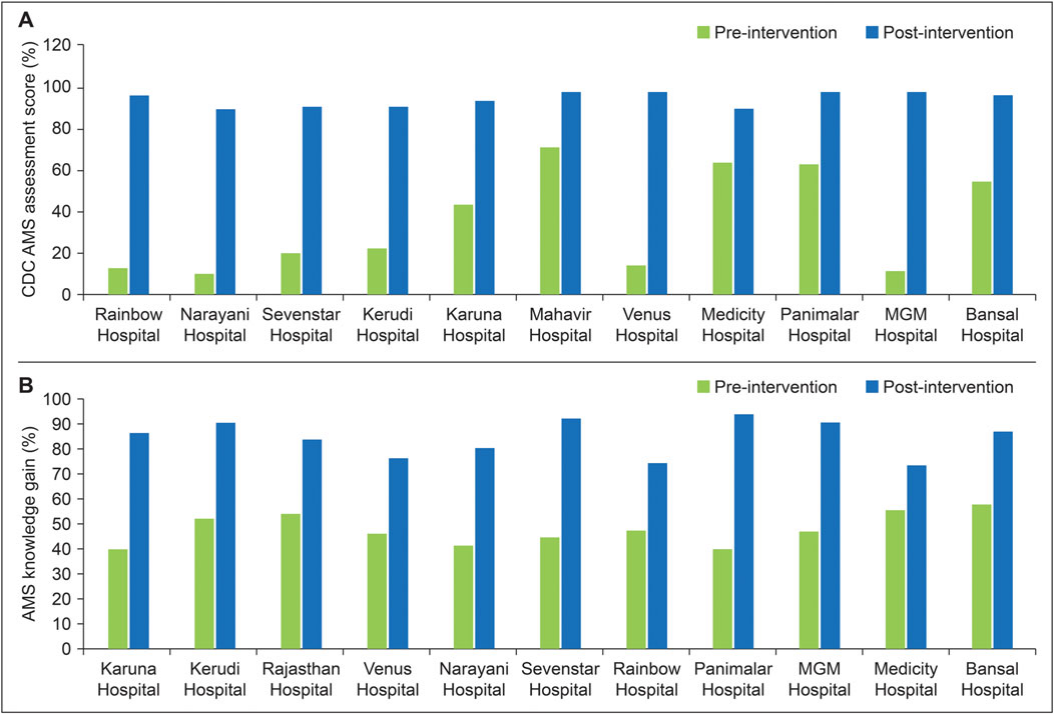
Figure 2 long description. (A) Improvement in CDC AMS assessment scores from the preintervention phase to the postintervention phase. (B) Improvements in knowledge regarding critical aspects of AMS. AMS, antimicrobial stewardship; CDC, Centers for Disease Control and Prevention.

Across all 11 hospitals, CDC AMS assessment scores increased significantly from a median of 22.2% (Q1–Q3: 13.2%–58.3%) at baseline to 95.8% (90.3%–97.2%) after the intervention (*P* = .0039, Figure 2A). Correspondingly, HCP knowledge improved, with median scores rising from 46.7% (42.6%–52.9%) to 86.4% (78.0%–90.0%) (*P* = .0039, Figure 2B), reflecting substantial gains in AMS practice and understanding.

### Compliance with antibiotic policy

Implementation of the AMSP across 11 hospitals substantially improved AMS practices. Antibiotic compliance, surgical prophylaxis, antibiotic selection, dose and duration optimization, IV-to-oral conversion, and de-escalation all showed significant gains from baseline to postintervention Table 1. Compliance rates increased from as low as 10% to over 80% in most hospitals, with surgical prophylaxis showing notable improvement at initially low-adherence sites. These findings highlight the effectiveness of AMSP interventions in optimizing antimicrobial use and patient care. Hospital-wise comparisons are shown in Table 2.

**Table 1.** Changes in AMS indicators from preintervention to postintervention stage Table 1 long description.

AMS indicator	Preintervention (median [Q1–Q3]) (%)	Postintervention (median [Q1–Q3]) (%)	*P* value*
Antibiotic compliance	61.0 (54.0**–**63.0)	86.0 (83.5**–**89.0)	.0038
Surgical prophylaxis	78.0 (59.0**–**87.5)	97.0 (94.5**–**99.5)	.0059
Antibiotic selection	61.0 (54.0**–**62.5)	86.0 (84.0**–**89.0)	.0038
Dose optimization	63.0 (57.5**–**75.0)	86.3 (79.5**–**93.0)	.0038
Duration optimization	55.0 (54.0**–**62.0)	86.0 (82.0**–**89.0)	.0038
Patients on IV but eligible for PO switch	30.0 (18.5**–**37.8)	13.5 (9.0**–**18.0)	.008
De-escalation	36.0 (22.5**–**66.0)	77.0 (65.5**–**88.5)	.0039

AMS, antimicrobial stewardship; IV, intravenous; PO, per os. *P* values calculated using the Wilcoxon signed-rank test.

**Table 2.** Compliance rates (%) for various AMS indicators in the preintervention and postintervention stages Table 2 long description.

		Karuna hospital	Narayani hospital	Seven star hospital	Rainbow hospital	Medicity hospital	Kerudi hospital	Venus hospital	Panimalar medical college	MGM hospital	Jaipuria mahavir hospital	Bansal hospital
Antibiotic compliance	Preintervention	59	61	61	52	10	64	47	56	63	63	71
	Postintervention	83	85	83	86	84	88	73	88	90	90	91
	PPC	24	24	22	34	74	24	26	32	27	27	20
Surgical prophylaxis	Preintervention	50	86	98	69	0	80	58	60	78	100	89
	Postintervention	82	100	99	97	98	95	81	97	94	100	100
	PPC	32	14	1	28	98	15	23	37	16	0	11
Antibiotic selection	Preintervention	59	61	61	52	10	64	48	56	62	63	71
	Postintervention	83	85	84	86	84	88	73	88	90	90	91
	PPC	24	24	23	34	74	24	25	32	28	27	20
Dose optimization	Preintervention	79	63	55	57	42	65	80	84	58	59	71
	Postintervention	100	84	80	72	79	90	86	96	79	98	89
	PPC	21	21	25	15	37	25	6	12	21	39	18
Duration optimization	Preintervention	53	67	55	57	45	69	53	55	55	57	71
	Postintervention	85	86	90	78	80	90	80	88	84	89	89
	PPC	32	19	35	21	35	21	27	33	29	32	18
Patient on IV therapy but eligible for PO switch	Preintervention	9	58	30	22	49	38	5	15	33	38	29
	Postintervention	10	35	14	22	21	14	4	8	15	6	11
	PPC	1	−23	−16	0	−28	−24	−1	−7	−18	−32	−18
De-escalation	Preintervention	27	36	18	29	18	50	84	61	11	82	71
	Postintervention	61	65	56	69	77	80	91	88	66	92	89
	PPC	34	29	38	40	59	30	7	27	55	10	18

AMS, antimicrobial stewardship; IV, intravenous; PO, oral administration; PPC, percentage point change.

### Antibiotic consumption

Implementation of the AMSP led to shifts in Access, Watch, and Reserve antibiotic use across hospitals (January–June 2023), Table 3. Use of Access antibiotics increased from baseline, though not significantly (*P* = .2664), indicating a move toward first-line therapy. Watch antibiotics decreased significantly (*P* = .0367), while Reserve antibiotic use declined without statistical significance (*P* = .3983).

**Table 3.** Changes in the prescription patterns of access, watch, and reserve antibiotic categories Table 3 long description.

	Access			Watch			Reserve		
Hospital	Preintervention (DDD/100 bed-days)	Postintervention (DDD/100 bed-days)	PPC	Preintervention (DDD/100 bed-days)	Postintervention (DDD/100 bed-days)	PPC	Preintervention (DDD/100 bed-days)	Postintervention (DDD/100 bed-days)	PPC
Karuna hospital	11.47	13.3	1.83	42.6	34.2	−8.4	16.8	6.5	−10.3
Narayani hospital	18.53	23.29	4.76	44.6	40.6	−4	4.21	5.84	1.63
Seven Star hospital	15.22	29.186	13.966	78.92	60.86	−18.06	5.44	4.5	−0.94
Rainbow hospital	51.61	62.86	11.25	27.98	10.11	−17.87	18.5	25.53	7.03
Medicity hospital	15	24	9	18	5	-13	23	2	−21
Kerudi hospital	4.07	1.74	−2.33	79.38	91.39	12.01	0.21	0.6	0.39
Venus hospital	20.6	18.59	−2.01	100.6	92.78	−7.82	2.12	0.89	−1.23
Panimalar medical college	12.5	11.31	−1.19	13.8	16.37	2.57	0.84	1.47	0.63
MGM hospital	33.8	26.12	−7.68	84.24	78.87	−5.37	3.24	0	−3.24
Jaipuria mahavir hospital	21.68	24.99	3.31	66.46	48.56	−17.9	27.6	12.75	−14.85
Bansal hospital	20.43	20.148	−0.282	42.17	41.077	−1.093	31.186	34.666	3.48
**Mean**	20.45	23.23	2.78	54.43	47.26	−7.18	12.1	8.61	−3.49
**SD**	12.15	14.62	6.51	27.72	29.51	9.47	11.03	10.89	8.43
**Median**	18.5	23.3	1.83	44.6	41.1	−7.82	5.4	4.5	−0.94
**IQR**	7.39	9.61	8.48	44.08	44.58	12.89	18.07	8.45	7.90

DDD, defined daily dose; IQR, interquartile range; PPC, percentage point change; SD, standard deviation.

### Incidence of MDROs

The implementation of the AMSP resulted in variable changes in the incidence rates of specific MDROs across the participating hospitals (January 23 to June 23). There was a reduction in the median incidence of MRSA and MDR nonfermenters Table 4. The overall change across hospitals was not statistically significant (*P* = .7557). Some hospitals observed increases in certain MDROs after the intervention.

**Table 4.** Incidence rates of specific MDROs per 1,000 inpatient admissions during the preintervention and postintervention stages Table 4 long description.

	MRSA			VRE			ESBL			CRE			MDR nonfermenters		
Hospital	Preintervention	Postintervention	PPC	Preintervention	Postintervention	PPC	Preintervention	Postintervention	PPC	Preintervention	Postintervention	PPC	Preintervention	Postintervention	PPC
Karuna hospital	6.2	1	−5.2	0	0	0	49.6	10	−39.6	10.9	4	−6.9	17.1	9	−8.1
Narayani hospital	3.2	4	0.8	0	0	0	6.4	12.1	5.7	0	8	8	9.6	16.2	6.6
Seven star hospital	1.8	1.5	−0.3	0	0	0	0	0	0	7.2	2.9	−4.3	10.8	19.1	8.3
Rainbow hospital	0	0	0	0	0	0	13.1	8.6	−4.5	1.1	1	−0.1	5.5	3.8	−1.7
Medicity hospital	0	0	0	3.3	1.3	−2	0	0	0	4.9	3.8	−1.1	4.9	2.5	−2.4
Kerudi hospital	0	0	0	0	0	0	4.2	1.6	−2.6	2.1	1.6	−0.5	6.4	4.8	−1.6
Venus hospital	0	3	3	0	1.5	1.5	8.7	13.6	4.9	2.5	1.5	−1	10	0	−10
Panimalar medical college	3	4.5	1.5	0	0	0	13	7.2	−5.8	4	3.3	−0.7	5	5.2	0.2
MGM hospital	6.1	0	−6.1	0	0	0	0	27	27	0	9	9	6.1	0	−6.1
Jaipuria mahavir hospital	1.2	0	−1.2	0	0	0	5.1	2.2	−2.9	6.4	8.8	2.4	14.2	9.9	−4.3
Bansal hospital	0.6	3	2.4	0.6	0	−0.6	0	1.2	1.2	0	0	0	20.2	27.8	7.6
**Mean**	2.01	1.55	−0.46	0.35	0.25	−0.10	9.10	7.59	−1.51	3.55	3.99	0.44	9.98	8.94	−1.05
**SD**	2.36	1.77	2.85	0.99	0.57	0.81	14.32	8.13	15.49	3.53	3.21	4.67	5.22	8.78	6.25
**Median**	1.20	1.00	0.00	0.00	0.00	0.00	5.10	7.20	0.00	2.50	3.30	−0.50	9.60	5.20	−1.70
**IQR**	3.10	3.00	1.90	0.00	0.00	0.00	10.85	9.65	6.75	5.10	4.45	2.25	6.70	9.90	8.60

CRE, carbapenem-resistant Enterobacteriaceae; ESBL, extended-spectrum beta-lactamase; IQR, interquartile range; MDR, multidrug-resistant; MDRO, multidrug-resistant organism; MRSA, methicillin-resistant Staphylococcus aureus; PPC, percentage point change; SD, standard deviation; VRE, vancomycin-resistant enterococci species.

## Discussion

This study aimed to design, implement, and evaluate a replicable AMS model suitable for healthcare facilities in resource-constrained settings, with the goal of developing a scalable framework for broader adoption. Implementing AMSPs in such settings is often challenged by the limited availability of local AMR data, the absence of standardized antimicrobial guidelines, and inadequate audit mechanisms.^22^ These constraints were considered in the study design, which focused on key elements to improve patient outcomes and safety while promoting judicious use of antimicrobials. However, the participating hospitals were not resource-naïve settings; site selection required leadership commitment, in-house microbiology capacity, and pharmacy systems capable of supporting antibiotic-use monitoring. Therefore, the improvements observed in this study likely reflect what can be achieved when structured AMS programming is paired with a minimum level of institutional infrastructure and support.

At baseline, AMS implementation varied across participating hospitals, as reflected by the range of CDC AMS assessment scores and variability in compliance indicators. Following antibiotic policy development, priority interventions included prospective audit and feedback, preauthorization of antibiotic use, and facility-specific treatment guidelines.^23^ Supplemental interventions, regular training, monitoring, and review helped sustain the initiative. The impact of AMSP was assessed using indicators of awareness, policy compliance, antibiotic consumption, and MDRO incidence across participating hospitals. Implementation of this AMS model resulted in significant KPI improvements, indicating its potential to enhance patient safety, optimize antimicrobial use, and improve clinical, microbiological, and economic outcomes.^24^

The study demonstrated substantial improvements in AMS practices, as reflected in higher CDC AMS assessment scores, highlighting effective integration of AMS protocols. These gains are particularly relevant given the slower adoption of AMSPs in India compared with that in Western countries.^25^ Focused educational initiatives significantly improved pre- and posttraining evaluations, enhancing HCP understanding of AMS concepts, a critical step, as such structured programs are scarce in India.^26,27^ Continuous training is essential to maintain and advance AMS skills, especially in resource-limited settings.^28^

Clinician adherence to antibiotic policies, particularly in surgical prophylaxis, improved markedly. Compliance gains in this study exceeded those reported in other Indian settings (61% and 77.7%).^29,30^ Interventions to promote policy adherence, including prescriber education and mobile applications, can enhance patient outcomes and reduce hospital stays.^31^ These findings highlight the importance of aligning clinical practices with hospital-specific antibiotic protocols. Continued efforts are needed to sustain and further improve compliance.^32^

A shift from “Reserve” to “Access” antibiotics, according to the WHO AWaRe classification, demonstrates the value of targeted AMS interventions, although further optimization is needed to maximize outcomes. Hospital-based AMSPs have been shown to reduce total antibiotic consumption by 19% and restricted antibiotic use by 27%,^33^ which is critical given India’s high infectious disease burden and antibiotic overuse.^3,34^ The current findings align with prior studies supporting AMSP implementation to optimize antibiotic use.^35–37^

Global antibiotic consumption rose between 2000 and 2015, particularly in low- and middle-income countries, highlighting the need for stewardship interventions.^38,39^ Heterogeneity in Reserve antibiotic use across hospitals may reflect local MDR outbreaks, clinician hesitancy to de-escalate, limited microbiological diagnostics, or institutional prescribing practices. Addressing these requires enhanced microbiology support, real-time prescription audits, and clinician training. Variability in MDRO incidence, including MRSA, VRE, ESBL, and MDR nonfermenters, may also reflect insufficient time for full program impact. Similar AMSPs, such as those in Qatar, achieved sustained reductions in MDR *Pseudomonas aeruginosa* and overall antimicrobial consumption.^38^ Comprehensive testing, equipped microbiology facilities, and clinician awareness are crucial for controlling MDROs in healthcare settings.^38^

Gaps in knowledge and in compliance with antimicrobial policies and guidelines observed before the intervention highlight ongoing challenges. There is a pressing need to scale up and refine AMSPs while addressing strategic barriers that limit their full potential globally.^22^ Existing frameworks, such as the National Quality Assurance Program and Kayakalp, can reinforce AMSPs in district and subdistrict hospitals.^11^ through infection control, standard treatment guidelines, prescription auditing, essential medicine lists, antimicrobial availability, and quality-based incentives. Strengthening opportunities include revising essential medicine lists per the WHO AWaRe classification, incorporating standard treatment guidelines from the WHO AWaRe antibiotic book and Indian Council of Medical Research guidelines, deploying dedicated AMS personnel, and conducting antimicrobial-specific prescription audits aligned with WHO and Indian Council of Medical Research recommendations.^11^

Implementing sustainable and effective AMSPs in low- and middle-income countries faces multiple challenges. Context-aware models tailored to local requirements are crucial for success in countries like India. Robust stewardship relies on committed teams, microbiologic support, and hospital information systems. Interventions successful in high-income settings may not translate directly to low-resource contexts and require adaptation.^40^ Evidence supporting scalable AMS strategies in low- and middle-income countries remains limited.^22^ Sustaining gains from optimized practices requires periodic monitoring and feedback. The positive results from this study contribute to resources that may facilitate broader AMS adoption in Indian healthcare settings.

A key limitation of this study was the lack of recorded patient illness severity, which may have influenced the analysis and interpretation. Limited documentation of preadmission interventions and antimicrobial use, particularly for referrals, affected data completeness. Hospital selection criteria, a small sample size, and the absence of a control group may limit generalizability. Variability in baseline parameters and intervention duration across hospitals could introduce unmeasured confounding. In addition, the follow-up period was relatively short, limiting assessment of the long-term sustainability. Interpretation of MDRO trends should also be cautious, as hospital-based AMS interventions may require longer follow-up and broader surveillance to demonstrate measurable effects on MDRO persistence, particularly in the context of referral patterns and catchment populations that were not assessed in this study.

### Future directions

To strengthen AMSPs in India, scalable, standardized models adaptable to diverse, resource-limited settings are needed.^22^ Establishing a national AMS data repository with standardized reporting would improve surveillance and benchmarking.^16^ Low- and middle-income-country-specific challenges should be addressed by integrating AMS into national health programs. Sustainability would require supportive institutional policies.

This study highlights the potential of well-designed AMSPs to reduce antimicrobial consumption and ensure policy compliance. Despite diverse challenges, the insights gained contribute to a valuable guide for implementing effective AMSPs, reinforcing the fight against AMR.

## Supporting information

10.1017/ash.2026.10430.sm001 Vasave and Paroha supplementary material 1 Vasave and Paroha supplementary material

10.1017/ash.2026.10430.sm002 Vasave and Paroha supplementary material 2 Vasave and Paroha supplementary material

## Supplementary material

The supplementary material for this article can be found at https://doi.org/10.1017/ash.2026.10430.

## Research transparency and reproducibility

The data sets generated and/or analyzed during the current study are not publicly available due to data sensitivity, as the supplementary files contain internal institutional documents. However, the data are available from the corresponding author on reasonable request.

## Figure 1. Long description

The flowchart outlines the phases of implementation for antimicrobial stewardship programs in hospitals. It begins with the Induction phase, lasting 2 to 0 months, which includes the end of process, selection criteria, selection committee, involvement of 11 hospitals across 7 states, and staff recruitment. This is followed by the Enrolment phase, lasting 1 to 9 months, where WHO and CDC tools are used, alignment with NCDC guidelines is ensured, NABH IPC-AMS assessment is conducted, and gaps are identified. The AMS assessment phase, lasting 10 to 11 months, involves setting 2 to 4 priorities per hospital and executing IPC capacity building and infrastructure, SOPs, and training of trainers, while facilitating IPC and AMS programs and recommending sustainability programming. The Implementation phase spans 12 to 32 months and includes the use of KAP tools for baseline and end line assessments, structural indicators based on the CDC checklist, and process and outcome measures. The Evaluation phase, lasting 33 to 34 months, focuses on report preparation, conferences, and events. The final phase is Dissemination, occurring at 35 to 36 months.

Navigate back to Figure 1..

## Figure 2. Long description

The bar graph consists of two sections labeled A and B. Section A compares CDC AMS assessment scores for various hospitals before and after an intervention. The x-axis lists hospitals such as Rainbow Hospital, Narayani Hospital, Sevenstar Hospital, and others. The y-axis represents the CDC AMS assessment score in percentage. Green bars indicate pre-intervention scores, while blue bars indicate post-intervention scores. All values are approximated. Section B compares AMS knowledge gain for the same hospitals before and after the intervention. The x-axis lists the same hospitals, and the y-axis represents the AMS knowledge gain in percentage. Green bars indicate pre-intervention knowledge gain, while blue bars indicate post-intervention knowledge gain. All values are approximated.

Navigate back to Figure 2..

## Table 1. Long description

The table presents data on AMS indicators, comparing preintervention and postintervention stages. It includes seven rows for different indicators: antibiotic compliance, surgical prophylaxis, antibiotic selection, dose optimization, duration optimization, patients on IV but eligible for PO switch, and de-escalation. Each row shows median percentages with interquartile ranges for preintervention and postintervention stages, along with P values indicating statistical significance. Notable improvements are observed in all indicators, with antibiotic compliance increasing from 61.0% to 86.0%, surgical prophylaxis from 78.0% to 97.0%, and de-escalation from 36.0% to 77.0%. The P values for all indicators are below 0.1, indicating significant improvements postintervention.

Navigate back to Table 1..

## Table 2. Long description

The table presents compliance rates for various antimicrobial stewardship (AMS) indicators across 11 hospitals, comparing preintervention and postintervention stages. The indicators include antibiotic compliance, surgical prophylaxis, antibiotic selection, dose optimization, duration optimization, patient on IV therapy but eligible for PO switch, and de-escalation. The table has 8 rows and 12 columns, including headers for each hospital and each indicator. Notable trends include significant improvements in compliance rates postintervention, with increases from as low as 10% to over 80% in most hospitals. Surgical prophylaxis shows notable improvement at initially low-adherence sites. The data highlights the effectiveness of AMSP interventions in optimizing antimicrobial use and patient care. Row 1: Antibiotic compliance, Preintervention, 59, 61, 61, 52, 10, 64, 47, 56, 63, 63, 71. Row 2: Antibiotic compliance, Postintervention, 83, 85, 83, 86, 84, 88, 73, 88, 90, 90, 91. Row 3: Antibiotic compliance, PPC, 24, 24, 22, 34, 74, 24, 26, 32, 27, 27, 20. Row 4: Surgical prophylaxis, Preintervention, 50, 86, 98, 69, 0, 80, 58, 60, 78, 100, 89. Row 5: Surgical prophylaxis, Postintervention, 82, 100, 99, 97, 98, 95, 81, 97, 94, 100, 100. Row 6: Surgical prophylaxis, PPC, 32, 14, 1, 28, 98, 15, 23, 37, 16, 0, 11. Row 7: Antibiotic selection, Preintervention, 59, 61, 61, 52, 10, 64, 48, 56, 62, 63, 71. Row 8: Antibiotic selection, Postintervention, 83, 85, 84, 86, 84, 88, 73, 88, 90, 90, 91. Row 9: Antibiotic selection, PPC, 24, 24, 23, 34, 74, 24, 25, 32, 28, 27, 20. Row 10: Dose optimization, Preintervention, 79, 63, 55, 57, 42, 65, 80, 84, 58, 59, 71. Row 11: Dose optimization, Postintervention, 100, 84, 80, 72, 79, 90, 86, 96, 79, 98, 89. Row 12: Dose optimization, PPC, 21, 21, 25, 15, 37, 25, 6, 12, 21, 39, 18. Row 13: Duration optimization, Preintervention, 53, 67, 55, 57, 45, 69, 53, 55, 55, 57, 71. Row 14: Duration optimization, Postintervention, 85, 86, 90, 78, 80, 90, 80, 88, 84, 89, 89. Row 15: Duration optimization, PPC, 32, 19, 35, 21, 35, 21, 27, 33, 29, 32, 18. Row 16: Patient on IV therapy but eligible for PO switch, Preintervention, 9, 58, 30, 22, 49, 38, 5, 15, 33, 38, 29. Row 17: Patient on IV therapy but eligible for PO switch, Postintervention, 10, 35, 14, 22, 21, 14, 4, 8, 15, 6, 11. Row 18: Patient on IV therapy but eligible for PO switch, PPC, 1, -23, -16, 0, -28, -24, -1, -7, -18, -32, -18. Row 19: De-escalation, Preintervention, 27, 36, 18, 29, 18, 50, 84, 61, 11, 82, 71. Row 20: De-escalation, Postintervention, 61, 65, 56, 69, 77, 80, 91, 88, 66, 92, 89. Row 21: De-escalation, PPC, 34, 29, 38, 40, 59, 30, 7, 27, 55, 10, 18.

Navigate back to Table 2..

## Table 3. Long description

The table presents data on the changes in prescription patterns of access, watch, and reserve antibiotic categories across various hospitals. It includes preintervention and postintervention values in defined daily doses per 100 bed-days, along with percentage point changes (PPC). The hospitals listed are Karuna hospital, Narayani hospital, Seven Star hospital, Rainbow hospital, Medicity hospital, Kerudi hospital, Venus hospital, Panimalar medical college, MGM hospital, Jaipuria mahavir hospital, and Bansal hospital. The table shows mean, standard deviation, median, and interquartile range values for each category. Notable trends include an increase in access antibiotics and a decrease in watch antibiotics post-intervention.

Navigate back to Table 3..

## Table 4. Long description

The table presents the incidence rates of specific multidrug-resistant organisms (MDROs) per 1,000 inpatient admissions across various hospitals during the preintervention and postintervention stages. It includes data for MRSA, VRE, ESBL, CRE, and MDR nonfermenters. The table has 13 rows and 17 columns, with hospitals listed in rows and different MDROs with their respective stages in columns. Notable trends include reductions in MRSA and MDR nonfermenters in some hospitals, while others observed increases in certain MDROs postintervention. The data highlights variable changes in incidence rates across participating hospitals.

Navigate back to Table 4..
