# Supplementary material for: Assessing the impact of antimicrobial stewardship in low-income healthcare settings: a study of antibiotic use and antimicrobial susceptibility patterns in Indian hospitals
Source: Antimicrob Steward Healthc Epidemiol. 2026 Jun 18;6(1):e181. doi: 10.1017/ash.2026.10430 (PMC13312239; doi:10.1017/ash.2026.10430)
Supplement: Vasave and Paroha supplementary material 1 — Vasave and Paroha supplementary material [file S2732494X26104306sup001.docx]

**Supplementary Information 1:** Sample pre- and post-training knowledge assessment questionnaires

**AMS Strategy and Surveillance**

1. **The core elements of Antimicrobial stewardship(AMS) includes all except:**
2. Action
3. Leadership
4. Responsibility
5. Education
6. **AMS Team structure includes all except:**
7. Microbiologist
8. Pharmacist
9. Accountant
10. ID physician
11. **Pharmacy based interventions of AMS includes all except:**
12. Dose adjustments
13. Dose optimization
14. Selective reporting of antimicrobial susceptibility testing results
15. Duplicative therapy alerts

1. **Microbiologist based interventions of AMS includes all except:**
2. Selective reporting of antimicrobial susceptibility testing results
3. Comments in microbiology reports
4. Duplicative therapy alerts
5. MIC interpretation notes in reports

1. **Provider based interventions of AMS includes all except:**
2. IV-oral conversion
3. Escalation-Descalation
4. Selective reporting of antimicrobial susceptibility testing results
5. Antibiotic timeout
6. Assessing penicillin allergy

1. **Key performance indicators(KPI) based on Outcome includes all except:**
2. AMR Rates
3. *Clostridium difficile* infection rate
4. Length of Stay (LOS)
5. Unexpected readmission rate
6. Antibiotic timeout
7. Antibiotic expenditure
8. **Key performance indicators (KPI) based on Process measures includes all except:**
9. Adherence to hospital guidelines
10. Defined Daily Dose (DDD)
11. Leadership
12. Days of Therapy (DOT)
13. Length of Therapy (LOT)
14. **Defined daily doses (DDD) means :**
15. The number of days that a patient receives atleast one dose of an antibiotic summed for each antibiotic
16. The total number of grams of an antibiotic purchased, dispensed or administered
17. Comparison of data before- and after- implementation of AMS program
18. The number of days a patient receives antibiotic therapy irrespective of the number of antibiotics administered

1. **Length of therapy (LOT) means:**
2. The number of days that a patient receives atleast one dose of an antibiotic summed for each antibiotic
3. The total number of grams of an antibiotic purchased, dispensed or administered
4. Comparison of data before- and after- implementation of AMS program
5. The number of days a patient receives antibiotic therapy irrespective of the number of antibiotics administered
6. **Days of therapy (DOT) means:**
7. The number of days that a patient receives atleast one dose of an antibiotic summed for each antibiotic
8. The total number of grams of an antibiotic purchased, dispensed or administered
9. Comparison of data before- and after- implementation of AMS program
10. The number of days a patient receives antibiotic therapy irrespective of the number of antibiotics administered
11. **The steps of sustainability of Antimicrobial stewardship(AMS) includes all except:**
12. Monitoring and assessing AMS program performance
13. Reporting AMS program performance
14. Comparison of data before- and after- implementation of AMS program
15. Modifying and adapting the AMS program
16. Continuing AMS education
17. **Types of AMS interventions includes all except:**
18. Educational
19. Persuasive
20. Leadership
21. Restrictive
22. Structural
23. **The Seven “R” of Antimicrobial stewardship(AMS) includes all except:**
24. Right drug
25. Right patient
26. Right dose
27. Right time
28. None of above
29. **Implementation of AMS program involves below steps:**
30. Perform a situation analysis
31. Assemble an AMS team
32. Select interventions
33. Select key performance indicators
34. Implement, educate and monitor

This steps can be arranged in sequence as follows:

Please select the correct sequence.

1. v-ii-iii-i-iv
2. i-iii-ii-v-iv
3. i-ii-iii-iv-v
4. ii-iii-iv-v-i
5. None of above
6. **The advantages of IV to Oral conversion includes all except:**
7. ease of administration
8. early discharge opportunities
9. decreased opportunities for hand hygiene
10. decreased IV-related adverse events
11. drug cost savings

**Total score:**

**Antimicrobial Resistance and**

**Antimicrobial susceptibility reports interpretation**

**Name:**

**Designation:**

**Department:**

**Date:**

1. **Enterococcus species are resistant to below antibiotics except:**
2. Aminoglycosides
3. Cephalosporins
4. Penicillins
5. Licosamides
6. **Antibiotics with Time dependent action includes all except:**
7. Penicillins
8. Cephaloporins
9. Carbapenems
10. Fluoroquinolones
11. **Antibiotics with Concentration dependent action includes all except:**
12. Aminoglycosides
13. Quinolones
14. Penicillins
15. Vancomycin
16. **Blood culture bottles collected for culture and sensitivity tests should be refrigerated:**
17. True
18. False
19. **Colistin should be tested for susceptibility by below method:**
20. Kirby bauer disc diffusion
21. MIC broth dilution
22. E test method
23. Agar dilution method

1. **Genotypic AST method includes all except:**
2. DNA array and microchips
3. PCR
4. Broth dilution method
5. loop-mediated isothermal amplification (LAMP) Unexpected readmission rate
6. **AST results interpretation includes all except:**
7. Sensitive
8. Resistant dose dependant
9. Intermediate
10. Resistant
11. **….. is defined as Minimum concentration of an antibiotic needed to inhibit visible growth of a single isolate of an organism.**
12. Minimum inhibitory concentration(MIC)
13. Minimum bacterial concentration (MBC)
14. Breakpoint
15. Efficacy point
16. **….. is discriminatory concentrations used in the interpretation of results of susceptibility testing to define isolates as susceptible, intermediate, or resistant**
17. Minimum inhibitory concentration(MIC)
18. Minimum bacterial concentration (MBC)
19. Breakpoint
20. Efficacy point

1. **Agencies that Determine Antimicrobial Breakpoints includes all except:**
2. CLSI
3. EUCAST
4. FDA
5. ICMR
6. **Breakpoints are determined using the following multidisciplinary approaches:**
   1. **Microbiological - Epidemiological cut-off**
   2. **Pharmacological - PK-PD index**
   3. **Clinical correlation**

**The correct sequence for setting breakpoints is:**

1. i – ii – iii
2. ii – I - iii
3. Ii – iii - i
4. Iii – I - ii
5. **……. is an ECV interpretive category defined by an Epidemiological cut-off (ECV) that describes isolates with no mechanisms of acquired resistance or reduced susceptibility for the antimicrobial agent being evaluated.**
6. Wild type
7. Non wild type
8. None of above

1. **……. is an an ECV interpretive category defined by an ECV that describes isolates with presumed or known mechanisms of acquired resistance and reduced susceptibility for the antimicrobial agent being evaluated**
   - 1. Wild type
     2. Non wild type
     3. None of above

1. **IF MIC “≤” Breakpoint YOU CAN USE THE DRUG with below Exceptions except:**
2. Drug doesn’t get to the site of action
3. Drug doesn’t achieve its goal pharmacodynamics parameters
4. Drug doesn’t have inducible resistance
5. Drug cost
6. None of above
7. **Epidemiological cut-off (ECV) indications includes:**
8. As a strong predictor of clinical response to therapy
9. When breakpoints has been published
10. Indicator of possible resistance mechanisms that could affect response to therapy
11. None of above

**Total score:**

**Essentials of Antibiogram and Antibiotic policy**

**Name:**

**Designation:**

**Department:**

**Date:**

1. **Bases of Antibiotic policy includes all except:**
2. Spectrum of antibiotic activity.
3. Pharmacokinetics /Pharmacodynamics.
4. Adverse effects.
5. Potential to select resistance.
6. None of above
7. **Aims of Antibiotic policy includes all except:**
8. Selection of patients for treatment
9. To have all antibiotics available
10. To increase costs of treatment
11. Avoid unnecessary antibiotic use
12. **Key elements of Antibiotic policy includes all except:**
13. Multidisciplinary committee
14. Recommended dose, duration and frequency of antibiotic
15. Auditing of the antibiotic prescriptions
16. Transmission based isolation precautions
17. **Hospital cumulative antibiogram is prepared using below document of CLSI:**
18. CLSI M39 A4
19. CLSI M39 B5
20. CLSI N39 C2
21. CLSI N39 A4
22. **Patient risk stratification is needed for antibiotic policy for below reasons:**
23. To decide narrow spectrum, broad or broadest spectrum
24. To increase patient costs
25. To identify healthcare associated infections
26. None of above
27. **CLSI Recommendation for Antibiotic policy preparation includes all except:**
28. Include only final verified test results.
29. ≥ 30 isolates.
30. Environmental surveillance isolates excluded.
31. Eliminate duplicates –only the first.
32. Only Resistance and not the intermediate.
33. **In patient risk stratification for treatment preference, patient category type I includes:**
34. Invasive fungal infections unlikely
35. Minimal risk of invasive fungal infections
36. Risk of fungal infections in immunocompromised and immunosuppressed
37. High risk of invasive fungal infections
38. **In** **patient risk stratification for treatment preference, patient category type II includes:**
39. Invasive fungal infections unlikely
40. Minimal risk of invasive fungal infections
41. Risk of fungal infections in immunocompromised and immunosuppressed
42. High risk of invasive fungal infections
43. **In patient risk stratification for treatment preference, patient category type III includes:**
44. Invasive fungal infections unlikely
45. Minimal risk of invasive fungal infections
46. Risk of fungal infections in immunocompromised and immunosuppressed
47. High risk of invasive fungal infections
48. **In patient risk stratification for treatment preference, patient category type IV includes:**
49. Invasive fungal infections unlikely
50. Minimal risk of invasive fungal infections
51. Risk of fungal infections in immunocompromised and immunosuppressed
52. High risk of invasive fungal infections
53. **Principle of Empirical Antibiotic therapy includes consideration of all except:**
54. Is the patient infectious
55. Which organ is sick
56. Which antibiotic should we use
57. What dose of antibiotic should we use
58. None of above
59. **Access group of antibiotics includes:**
60. Amikacin
61. Meropenem
62. Colistin
63. None of above

1. **Watch group of antibiotics includes:**
2. Amikacin
3. Meropenem
4. Colistin
5. None of above

1. **Reserve group of antibiotics includes:**
2. Amikacin
3. Meropenem
4. Colistin
5. None of above
6. **Surgical prophylaxis antibiotic timing includes:**
7. One hour before incision of surgery
8. One hour after incision of surgery
9. three hours before incision of surgery
10. three hours after incision of surgery

**Total score:**

**KPIs of Antibiotic Policy**

**Name:**

**Designation:**

**Department:**

**Date:**

1. **Antimicrobial stewardship covers**
2. Dose optimization
3. Limiting inappropriate use of antimicrobials.
4. Limiting cost of therapy
5. All of the above
6. **Quantity measures of AMS are**
7. Defined daily dose
8. Multidrug resistance
9. Surgical prophylaxis compliance
10. Number of new infections
11. **Find the correct statement**
12. Dose adjustment should be done for all patients irrespective of patient condition.
13. Age, sex, height are the basic parameters considered for dose adjustment.
14. Cockcroft – Gault equation is used for renal dose adjustment.
15. Cockcroft – Gault equation is same for all gender.
16. **Antibiotic dose depends on**
17. Causative organisms
18. Type of Formulation
19. Type of infection
20. All of the above
21. **Find the wrong statement**
22. Adjusted body weight= IBW+0.4(Actual body weight-Ideal body weight)
23. Vancomycin dose calculation is based on total body weight
24. Ideal body weight and actual body weight are same.
25. Daptomycin dose calculation is based on adjusted body weight.
26. **Pick the correct statement,**
27. Loading dose is an initial higher dose of a drug that may be given at the beginning of course of treatment.
28. Dose given to maintain steady state concentration
29. Loading dose of colistin is 3mu .
30. All of the above
31. **Selection of empiric therapy depends on**
32. diagnosis
33. host organism
34. hospital antibiogram
35. all of the above
36. **Process indicators include all except**

a)De-escalation

b)Duration of agent

c)Days of therapy

d)Optimizing dose.

1. **Antimicrobials which show excellent bioavailability in both iv and oral forms are all except,**

a) Metronidazole

b) Doxycycline

c) Vancomycin

d) Linezolid

1. **Restricted antimicrobial includes all except**

a) Colistin

b) Tigecycline

c) Ciprofloxacin

d) Caspofungin

**Total score:**

**MDRO**

**Name:**

**Department:**

**Designation:**

**Date:**

**1) Clinical Factors associated with MDROS includes all except:**

1. Increased length of stay
2. Increased use of Antibiotics
3. Less treatment cost
4. Mortality rate

**2) The most challenging MDROS in healthcare System includes:**

1. Methicillin-resistant staphylococcus aureus (MRSA)
2. Vancomycin-resistant enterococcus (VRE)
3. Extended-spectrum beta lactamase producing bacteria(ESBL)
4. All of the above

**3) What ways by which a bacteria could become resistant to carbapenems:**

1. Production of carbapenemases
2. Structural mutations and coupled activity
3. Efflux pumps and porin mutations
4. All of the above

**4) Risk Factors Contributing to MRSA infection for all facility types includes all except:**

1. High Functional status
2. Presence of invasive devices
3. History of colonization
4. Prior antimicrobial therapy

**5) Vancomycin –Resistant Enterococcus (VRE) are resistant to below antibiotic classes except:**

1. Penicillin’s
2. Aminoglycosides
3. Cephalosporins
4. Carbapenems

**6) Steps to prevent Antimicrobial Resistance for hospitalized adults:**

1. Vaccinate
2. Get the catheters out
3. Access the expert
4. Practice antimicrobial control
5. Break the chain of contagion

The correct sequence for preventing antimicrobial resistance

1. 1,2,3,4,5
2. 1,3,5,4,2
3. 2,3,4,5,1
4. 5,4,3,2,1

**7) Prevention and control strategies of MDRO transmission include all except:**

1. Improvements in hand hygiene
2. Education
3. Inactive Surveillance
4. Use of contact precautions until patients are culture negative

**8) Disadvantages of vancomycin for the treatment of MRSA includes all except:**

1. Parenteral administration
2. inexpensive
3. Ototoxicity
4. Can potentiate nephrotoxicity of aminoglycosides

**9) ESBL is resistant towards following antibiotics except:**

1. Penicillin’s
2. 1^st^ and 2^nd^ Cephalosporins
3. Aztreonam
4. Carbapenems

**10) is MDRO transmitted by airborne/Contact /Droplet?**

a) MDRO transmitted by different routes, including blood borne, droplet, air borne and droplet

b) Contact

c) Air borne

d) Droplet

**Total score:**

**PRINCIPLES OF ANTIBIOTIC THERAPY**

**1) Choose the correct set of statements**

1. Bacteriostatic antibiotics prevent replication of bacteria
2. Bacteriostatic antibiotics kill the bacteria
3. Bactericidal antibiotics kill the bacteria
4. Bactericidal antibiotics prevent replication of bacteria

a) i b) iii c) i & iii d) ii & iv

**2) Choose the correct set of statements**

1. PK refer to absorption, metabolism, distribution, and elimination
2. PD refer to the effects of the drug on the body or organism
3. PK refer to the effects of the drug on the body or organism
4. PD refer to absorption, metabolism, distribution, and elimination

a) i & ii b) i alone c) ii alone d) iii & iv

**3) The following are concentration dependant antibiotics except**

1. Aminoglycosides
2. Fluoroquinolones
3. Beta Lactams
4. Colistin

**4) The following are time dependant antibiotics except**

1. Erythromycin
2. Linezolid
3. Clindamycin
4. Metronidazole

**5) Criteria for selecting an antimicrobial regimen includes all except**

1. Agent should be active against the (expected) pathogen
2. Should have an appropriately narrow spectrum
3. Concentration should be the minimum at the site of infection
4. Should have the least toxicity and least costs when compared with equally effective drugs

**6) Empirical treatment of CAP with no structural lung pathology could be all except**

1. Amoxicillin + Clavulanate 1.2gm BD
2. Ceftriaxone 1gm BD + Azithromycin 500mg OD
3. Piperacillin + Tazobactam 4.5gm TID + Azithromycin 500mg OD
4. Ceftriaxone 1gm BD + Doxycycline 100mg BD

**7) Criteria for considering Per Oral except**

1. Vital signs should be good
2. Oral intake should be possible.
3. Good bioavailability
4. Absence of malabsorption
5. Ongoing sepsis

**8) Criteria period is**

1. First 48 hours from the point of diagnosis
2. First 8 hours
3. Second 24 hours
4. First 72 hours

**9) Antibiotics dose depends on**

1. Site of Infection
2. Causative organisms
3. Type of Formulation
4. All the above

**10) Route, compatibility, dilution details, final concentration, and infusion time are key factors for successful therapy**

1. True
2. False

**Total score:**
