# Supplementary material for: Assessing the impact of antimicrobial stewardship in low-income healthcare settings: a study of antibiotic use and antimicrobial susceptibility patterns in Indian hospitals
Source: Antimicrob Steward Healthc Epidemiol. 2026 Jun 18;6(1):e181. doi: 10.1017/ash.2026.10430 (PMC13312239; doi:10.1017/ash.2026.10430)
Supplement: Vasave and Paroha supplementary material 2 — Vasave and Paroha supplementary material [file S2732494X26104306sup002.docx]

| \|  \| \| --- \| | 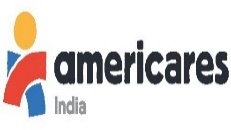 |  |  |  |  |  |  |  |  |  |  |  |  |  |  |  |  |  |  |  |  |  |  |  |  |
| --- | --- | --- | --- | --- | --- | --- | --- | --- | --- | --- | --- | --- | --- | --- | --- | --- | --- | --- | --- | --- | --- | --- | --- | --- | --- | --- |
|  |  |  |  |  |  |  |  |  |  |  |  |  |  |  |  |  |  |  |  |  |  |  |  |  |  |
|  |  |  |  |  |  |  |  |  |  |  |  |  |  |  |  |  |  |  |  |  |  |  |  | AIF/AMS/APC/22/2022 | |
| **KPI- Compliance to Antibiotic Policy** | | | | | | | | | | | | | | | | | | | | | | | |  |  |
| **Name of Hospital** | | |  |  |  |  |  |  |  |  |  |  |  |  |  |  |  |  |  |  |  |  |  |  |  |
|  | UHID no. | Age/Sex | Indication Diagnosis | Empirical Therapy given (Y/N) | Antibiotic Name Given | Started on | Dose | Route IV IM ORAL | Interval- OD, BD | Days- Duration | Last Dose Date | Appropriate Culture sent before starting (Y/N) | Specimen | Name of the organisum | Antibiotic changed after culture report (Y/N) | Antibiotic Review -24hrs | De-esclation done (Y/N) | De-escalated antibiotic name and dose | Is Antibiotic in reserve /Restricted category) | Reserve Drug policy followed(y/n) | Selection of Antibiotics as per Exsiting policy or Standard Treatjment guidelines( Y/N) | Policy compliance (Y/N) | % Compliance | MDRO/VRE/MRSA | Remarks |
|  |  |  |  |  |  |  |  |  |  |  |  |  |  |  |  |  |  |  |  |  |  |  |  |  |  |
| 1 |  |  |  |  |  |  |  |  |  |  |  |  |  |  |  |  |  |  |  |  |  |  |  |  |  |
| 2 |  |  |  |  |  |  |  |  |  |  |  |  |  |  |  |  |  |  |  |  |  |  |  |  |  |
| 3 |  |  |  |  |  |  |  |  |  |  |  |  |  |  |  |  |  |  |  |  |  |  |  |  |  |
| 4 |  |  |  |  |  |  |  |  |  |  |  |  |  |  |  |  |  |  |  |  |  |  |  |  |  |
| 5 |  |  |  |  |  |  |  |  |  |  |  |  |  |  |  |  |  |  |  |  |  |  |  |  |  |
| 6 |  |  |  |  |  |  |  |  |  |  |  |  |  |  |  |  |  |  |  |  |  |  |  |  |  |
| 7 |  |  |  |  |  |  |  |  |  |  |  |  |  |  |  |  |  |  |  |  |  |  |  |  |  |
| 8 |  |  |  |  |  |  |  |  |  |  |  |  |  |  |  |  |  |  |  |  |  |  |  |  |  |
| 9 |  |  |  |  |  |  |  |  |  |  |  |  |  |  |  |  |  |  |  |  |  |  |  |  |  |
| 10 |  |  |  |  |  |  |  |  |  |  |  |  |  |  |  |  |  |  |  |  |  |  |  |  |  |

**Supplementary Information 2:** Sample compliance audit tools

|  | | | | |  | | | | | | | | | | | | |  | | | | | |  | | | | | | |  | | | |  | | | | | AIF/AMS/AAT/23/2022 | | | | | |  | | | |
| --- | --- | --- | --- | --- | --- | --- | --- | --- | --- | --- | --- | --- | --- | --- | --- | --- | --- | --- | --- | --- | --- | --- | --- | --- | --- | --- | --- | --- | --- | --- | --- | --- | --- | --- | --- | --- | --- | --- | --- | --- | --- | --- | --- | --- | --- | --- | --- | --- | --- |
| **AMS KPI** | | | | | | | | | | | | | | | | | | | | | | | | | | | | | | | | | | | | | | | | | | | | | | | | | |
|  | | | | |  | | | | | | | | | | | | | **Prescribing indicators/KPIs;** | | | | | | | | | | | | | | | | | | | | | | | | | | | | | | | |
|  | | | | |  | | | | | | | | | | | | | **Target** | | | | | | **W1** | | | | | | | **W2** | | | | **W3** | | | | | **W4** | | | | | | **M1** | | | |
|  | | | | |  | | | | | | | | | | | | | % | | | | | | % | | | | | | | % | | | | % | | | | | % | | | | | | % | | | |
| **1** | | | | | **Compliance of choice of agent with Antibiotic policy** | | | | | | | | | | | | |  | | | | | |  | | | | | | |  | | | |  | | | | |  | | | | | |  | | | |
| **2** | | | | | **Dose Optimization** | | | | | | | | | | | | |  | | | | | |  | | | | | | |  | | | |  | | | | |  | | | | | |  | | | |
| **3** | | | | | **Antibiotic review 24hours** | | | | | | | | | | | | |  | | | | | |  | | | | | | |  | | | |  | | | | |  | | | | | |  | | | |
| **4** | | | | | **% of antibiotic Switch from one class to another class** | | | | | | | | | | | | |  | | | | | |  | | | | | | |  | | | |  | | | | |  | | | | | |  | | | |
| **5** | | | | | **% of antibiotics continued after culture report** | | | | | | | | | | | | |  | | | | | |  | | | | | | |  | | | |  | | | | |  | | | | | |  | | | |
| **6** | | | | | **Compliance of duration of agent with local policy/stopping of antibiotic** | | | | | | | | | | | | |  | | | | | |  | | | | | | |  | | | |  | | | | |  | | | | | |  | | | |
| 7 | | | | | **Conversion from broad spectrum to narrow spectrum** | | | | | | | | | | | | |  | | | | | |  | | | | | | |  | | | |  | | | | |  | | | | | |  | | | |
| **8** | | | | | **Patient on IV therapy but eligible for PO switch** | | | | | | | | | | | | |  | | | | | |  | | | | | | |  | | | |  | | | | |  | | | | | |  | | | |
| 9 | | | | | **De-escalation rate** | | | | | | | | | | | | |  | | | | | |  | | | | | | |  | | | |  | | | | |  | | | | | |  | | | |
| 10 | | | | | **Number of prescriptions with single antibiotic** | | | | | | | | | | | | |  | | | | | |  | | | | | | |  | | | |  | | | | |  | | | | | |  | | | |
| 11 | | | | | **Number of prescriptions with multiple antibiotic** | | | | | | | | | | | | |  | | | | | |  | | | | | | |  | | | |  | | | | |  | | | | | |  | | | |
| **12** | | | | | **% of restricted antimicrobial agents compliant with restricted use guidelines** | | | | | | | | | | | | |  | | | | | |  | | | | | | |  | | | |  | | | | |  | | | | | |  | | | |
|  | | | | |  | | | | | | | | | | | | |  | | | | | |  | | | | | | |  | | | |  | | | | |  | | | | | |  | | | |
| **13** | | | | | **Documentation of indication** | | | | | | | | | | | | |  | | | | | |  | | | | | | |  | | | |  | | | | |  | | | | | |  | | | |
| **14** | | | | | **Compliance of Surgical prophylaxis choice** | | | | | | | | | | | | |  | | | | | |  | | | | | | |  | | | |  | | | | |  | | | | | |  | | | |
| **15** | | | | | **Surgical prophylaxis duration/% of surgical prophylaxis prescriptions with duration <= 1day** | | | | | | | | | | | | |  | | | | | |  | | | | | | |  | | | |  | | | | |  | | | | | |  | | | |
| **16** | | | | | **% of surgical prophylaxis prescriptions with duration > 1day** | | | | | | | | | | | | |  | | | | | |  | | | | | | |  | | | |  | | | | |  | | | | | |  | | | |
| **17** | | | | | **% of cases who recevied appropriate prophylactic antibiotics with in specified time frame** | | | | | | | | | | | | |  | | | | | |  | | | | | | |  | | | |  | | | | |  | | | | | |  | | | |
|  | | | | |  | | | | | | | | | | | | |  | | | | | |  | | | | | | |  | | | |  | | | | |  | | | | | |  | | | |
| **18** | | | | | **Multidrug resistant organisms(MDROs)** | | | | | | | | | | | | |  | | | | | |  | | | | | | |  | | | |  | | | | |  | | | | | |  | | | |
|  | |  | | | |  | | |  | | |  | | |  | | | |  | | |  | | | | |  | | |  | | | |  | | | |  | | | **AIF/IPC/SPST/09/2021** | | |  | | | |  | |
|  |  | | |  | | | |  | | |  | | |  | |  | | | |  | | |  | | |  | | |  | | | |  | | |  | | |  | | |  | | |  | | | |  |
| **S.No.** | | | | | | | | | | | | | | | | | | | | | | | | | **SSI PROPHLYAXIS SURVEILLANCE TOOL (Month- ------------------------)** | | | | | | | | | | | | | | | | | | | | | | | | |
|  | | | **Name of Hospital** | | | |  | | |  | | |  | | | |  | | | |  | | | |  | | |  | | | |  | | | | |  | | |  | | |  | | | |  | | |
|  | **Date of surgery** | | | **Patient ID** | | | | **Age/Sex (M/F)** | | | **Name of surgery** | | | **Time of incision** | | **Time of antibiotic** | | | | **Duration of sugery** | | | **Name of antibiotic** | | | **Time difference bet. Incision and andtibiotic administration** | | | **Whether antibiotic was repeated/Redosing after 4 hours** | | | | **How long antibioticwas continued/Therapeutic** | | | **Telephonic/OPD follow up Date** | | | **Compliance -Yes / No** | | | **% Compliance** | | | **Data collected by CP** | | | | **Data Verified by ICMO** |
| 1 |  | | |  | | | |  | | |  | | |  | |  | | | |  | | |  | | |  | | |  | | | |  | | |  | | |  | | |  | | |  | | | |  |
| 2 |  | | |  | | | |  | | |  | | |  | |  | | | |  | | |  | | |  | | |  | | | |  | | |  | | |  | | |  | | |  | | | |  |
| 3 |  | | |  | | | |  | | |  | | |  | |  | | | |  | | |  | | |  | | |  | | | |  | | |  | | |  | | |  | | |  | | | |  |
| 4 |  | | |  | | | |  | | |  | | |  | |  | | | |  | | |  | | |  | | |  | | | |  | | |  | | |  | | |  | | |  | | | |  |
| 5 |  | | |  | | | |  | | |  | | |  | |  | | | |  | | |  | | |  | | |  | | | |  | | |  | | |  | | |  | | |  | | | |  |
| 6 |  | | |  | | | |  | | |  | | |  | |  | | | |  | | |  | | |  | | |  | | | |  | | |  | | |  | | |  | | |  | | | |  |
| 7 |  | | |  | | | |  | | |  | | |  | |  | | | |  | | |  | | |  | | |  | | | |  | | |  | | |  | | |  | | |  | | | |  |
| 8 |  | | |  | | | |  | | |  | | |  | |  | | | |  | | |  | | |  | | |  | | | |  | | |  | | |  | | |  | | |  | | | |  |
| 9 |  | | |  | | | |  | | |  | | |  | |  | | | |  | | |  | | |  | | |  | | | |  | | |  | | |  | | |  | | |  | | | |  |
| 10 |  | | |  | | | |  | | |  | | |  | |  | | | |  | | |  | | |  | | |  | | | |  | | |  | | |  | | |  | | |  | | | |  |

| This document is adapted by Americares India Foundation |
| --- |
| Reference –Centres for disease control and prevention |
| To be filled by Clinical Pharmacist |
